# Dietary Flavonoids and Insulin Signaling in Diabetes and Obesity

**DOI:** 10.3390/cells10061474

**Published:** 2021-06-11

**Authors:** María Ángeles Martín, Sonia Ramos

**Affiliations:** 1 Department of Metabolism and Nutrition, Institute of Food Science and Technology and Nutrition (ICTAN-CSIC), José Antonio Novais 10, Ciudad Universitaria, 28040 Madrid, Spain; amartina@ictan.csic.es; 2 Spanish Biomedical Research Centre in Diabetes and Associated Metabolic Disorders (CIBERDEM), Instituto de Salud Carlos III (ISCIII), 28040 Madrid, Spain

**Keywords:** dietary flavonoids, type 2 diabetes, obesity, insulin signaling, cultured cells, pre-clinical studies, human clinical trials

## Abstract

Type 2 diabetes (T2D) and obesity are relevant worldwide chronic diseases. A common complication in both pathologies is the dysregulation of the insulin-signaling pathway that is crucial to maintain an accurate glucose homeostasis. Flavonoids are naturally occurring phenolic compounds abundant in fruits, vegetables and seeds. Rising evidence supports a role for the flavonoids against T2D and obesity, and at present, these compounds are considered as important potential chemopreventive agents. This review summarizes *in vitro* and *in vivo* studies providing data related to the effects of flavonoids and flavonoid-rich foods on the modulation of the insulin route during T2D and obesity. Notably, few human studies have evaluated the regulatory effect of these phenolic compounds at molecular level on the insulin pathway. In this context, it is also important to note that the mechanism of action for the flavonoids is not fully characterized and that a proper dosage to obtain a beneficial effect on health has not been defined yet. Further investigations will contribute to solve all these critical challenges and will enable the use of flavonoids to prevent, delay or support the treatment of T2D and obesity.

## 1. Introduction

Current life style has led to an enhanced prevalence of important metabolic diseases such as type 2 diabetes (T2D) and obesity. Actually, both pathologies are considered as the most common chronic diseases in nearly all countries and constitute an international health burden [1,2]. According to the World Health Organization (WHO), the number of diabetic subjects has risen from 108 million in 1980 to 422 million in 2014 [1], and the incidence of obesity has nearly tripled since 1975 [2].

The pathogenesis of T2D and obesity is very different, but dysregulation of the insulin signaling pathway, which is present in T2D, is also a common complication in obesity; indeed, accumulating evidence suggests that obesity increases the risk for developing insulin resistance and T2D, among other pathologies [3]. Under these conditions of altered insulin signaling, the glucose homeostasis is dysregulated and the main peripheral organs involved in this systemic glucose dynamic are affected, i.e., mainly liver, adipose tissue and skeletal muscle [3,4]. It is important to note that current drugs are not satisfactorily effective in maintaining a long-term glycemia control in most patients. Thus, at present, it is considered that the most efficient approach to prevent or delay T2D and obesity is the reduction of sedentarism and changes in dietary habits. In this regard, flavonoids, which are natural dietary compounds abundant in vegetables and fruits, have attracted a great interest because of their lack of toxicity and potential ability to act as highly effective chemopreventive agents against T2D and obesity [5,6]. The aim of the present review is summarizing the molecular basis of the chemopreventive activity of flavonoids related to insulin signaling during T2D and obesity. In addition, the scarcely current existing evidence on the link between these natural compounds and insulin sensitivity based on human clinical trials is described.

## 2. Insulin Signaling, Diabetes and Obesity

Under physiological conditions, the circulating glucose is transported into β-pancreatic cells of the islets of Langerhans by the glucose transporter (GLUT)-2, leading to insulin secretion [7]. Then, the hormone binds to its specific cell surface receptor (insulin receptor, IR) and the insulin signaling route is activated. This stimulation leads to the phosphorylation of the insulin receptor substrates (IRS)-1 and -2, which is associated with the activation of both the phosphatidylinositol 3-kinase (PI3K)-AKT/protein kinase B (PKB) pathway and the Ras-mitogen-activated protein kinase (MAPK) route [7,8]. The PI3K/AKT pathway is important for the most metabolic actions of insulin. Tyrosine phosphorylated IRS-1 binds and activates the lipid kinase PI3K that then stimulates a serine/threonine kinases cascade, including AKT. Ultimately, the activation of this pathway results in an enhanced translocation of the insulin-responsive GLUTs to the plasma membrane, and the increased glucose uptake in the skeletal muscle and adipose tissue [3]. In the liver, the stimulation of the PI3K/AKT route leads to the activation of glycogen synthesis and the suppression of gluconeogenesis [3,7]. In addition, the MAPK pathway is important for the effects of insulin on cell growth (mitogenesis, cell differentiation, motility and survival), and it is not involved in mediating the metabolic actions of the hormone [8].

In view of all this, it becomes clear that the tight regulation of the insulin signaling is crucial for maintaining the glucose homeostasis and health [3,4,7,8]. Insulin-mediated signaling controls the glycemia by coordinating the production of glucose in the liver through the glycogenolysis and gluconeogenesis during fasting, and with its uptake in feeding times into the skeletal muscle through the glycogen synthesis and glucose metabolism, and to a much lesser extent into the adipose tissue [3,4,7]. Indeed, alterations in the insulin secretion and signaling lead to an imbalanced metabolism that predisposes to different diseases [4,8]. Thus, the malfunction of β-cells through oxidative stress and the impaired response of peripheral tissues to insulin (insulin resistance) lead to a situation of hyperglycemia and hyperinsulinemia together with a chronic low-grade of inflammation [3,4]. All these alterations are present in T2D, which is characterized by the declining of the β-cell function and worsening of insulin resistance [3]. In addition, insulin resistance is a hallmark of obesity, and it has been suggested that unbalanced lipid metabolism, dysbiosis, chronic inflammation and dysregulation of signaling pathways (insulin route) contribute to the development of the insulin resistance in this disease [3,4]. Moreover, a connection between obesity and T2D has been demonstrated [3].

During insulin resistance, the earliest defect is that, in the insulin signaling pathway, the autophosphorylation of IR is less responsive to the hormone [3,4,8]. Consequently, the downstream cellular action of insulin is seriously reduced or impaired. In addition, during insulin resistance in the skeletal muscle and adipose tissue, the glucose uptake is impaired because of the decreased AKT activity that leads to diminished GLUT-4 expression and translocation [3]; thus, the reduced AKT levels are connected with a decreased glycogen synthesis in the skeletal muscle [3]. Similarly, during insulin resistance, the hepatic AKT activity declines and that leads to both upregulation of forkhead box protein O1 (FOXO1) and promotion of gluconeogenesis, as well as to the diminution of glycogen synthesis [3]. Importantly, the accumulation of visceral adipose tissue leads to an excessive release of free fatty acids (FFA), which interferes with the insulin signaling by promoting protein kinases such as protein kinase C (PKC), MAPK, c-Jun N-terminal kinase (JNK) and inhibitor of nuclear factor κB kinase β (IκB-β) [3,4]. In addition, the low-grade of inflammation associated with the situation of insulin resistance results in an enhanced production and secretion of pro-inflammatory mediators (tumor necrosis factor [TNF]-α, interleukin [IL]-6, etc.) that in turn inhibits the insulin signaling pathway [3,4,7].

As mentioned above, insulin signaling alteration is a common hallmark in T2D and obesity. Both pathologies are recognized as the most common chronic diseases in almost all countries, and constitute an increasing international health burden [1,2]. Therefore, there is an urgent need to continue working on the prevention and control of these diseases, being the dietary interventions a very promising and economic approach. In line with this, flavonoids, which are natural compounds, have been receiving a rising interest.

## 3. Dietary Flavonoids

Flavonoids are plant secondary metabolites widely distributed in fruits, vegetables and seeds, as well as in their derived products such as cocoa, coffee, tea, soy-based foods and red wine [9]. Plants synthesize flavonoids for their protection against microbial invasion, oxidation injury and UV damage; in addition, they account for the odors, color and taste of foods. Structurally, flavonoids are included into the group of phenolic compounds as they have a basic polyphenolic structure consisting of two benzene rings (A and B) connected by an oxygenated heterocyclic ring (C) (Table 1) [10]. Depending on the functional groups present on the C-ring (methyl, hydroxyl, glycan, acetyl or others), the degree of C-ring oxidation and the connection position of B-ring, flavonoids are classified into six different subclasses, namely flavones, flavanones, flavonols, flavanols, isoflavones and anthocyanidins (Table 1). At the same time, individual compounds from each subclass are structurally distinguished by different patterns of hydroxylation and conjugation of the phenolic rings. However, flavonoids also occur as oligomers and polymers (i.e., tannins), and are classified as condensed tannins (also known as proanthocyanidins or procyanidins) or hydrolysable tannins [10]. Most flavonoids are present in plants as a glycoside-bound form, which contributes to their complexity and the large number of individual molecules that have been identified. Indeed, more than 9000 flavonoids have been described [11], even though, at present, compounds belonging to this group are still being identified.

Although the consumption of flavonoids through the diet may be different among populations, it has been estimated that their average total intake in Western countries is approximately 435 mg/day and may even increase in people with diets rich in plant-based foods [12,13]. However, the biological activity of flavonoids not only depend on their intake, but also on their bioavailability [14,15]. Once ingested, the absorption, metabolism and excretion of flavonoids in the human body determine their potential bioactivity. In brief, in the small intestine, flavonoids are metabolized and generate sulfates, glucuronides, and methylated metabolites, which are more soluble in water. Then, the conjugated metabolites pass to the portal vein and liver, where they undergo further phase II metabolism before appearing into the blood and being excreted in the urine [14]. Certain plasma metabolites can also be secreted in the bile to the duodenum and can be reabsorbed, increasing their half-life in the systemic circulation. In general, pure flavonoids and its conjugated forms are detected in plasma at nM or low µM concentrations after the regular consumption of flavonoids or flavonoid-rich foods [15]. In addition, the colon plays an important role in the bioavailability of dietary flavonoids, since a relatively high amount of these natural compounds is not absorbed in the small intestine. These unabsorbed flavonoids pass unaltered to the large intestine, where they are extensively metabolized by the microbiota into a variety of small phenolic acids and aromatic compounds that can easily be absorbed in the colon [16]. Interestingly, these derived colonic metabolites (mainly phenylpropionic, phenylacetic and benzoic acid derivatives) can be found in plasma at higher concentrations (µM levels) than those of pure flavonoids and their conjugated forms [17,18]. In consequence, parent compounds and their metabolites, as well as the colonic metabolites generated by the gut microbiota are considered the main contributors to the biological activities of flavonoids [16].

Scientific interest in flavonoids has considerably increased in recent decades with numerous studies supporting their beneficial effects on metabolic diseases such as T2D and obesity [6,19,20]. These natural compounds and their metabolites possess a number of biological functions such as antioxidant and anti-inflammatory activities that confer them numerous health-promoting properties [21]. Moreover, flavonoids have the ability to directly interact with proteins such as key cellular receptors or components of signaling pathways, thus affecting numerous functions in different cells and tissues [22]. Accordingly, flavonoids can reduce insulin resistance in insulin-sensitive tissues through various mechanisms, including the regulation of the insulin signaling pathway.

Under physiological and pathological situations, these natural compounds are able to stimulate the secretion of insulin from the β-pancreatic cells and activate the insulin signaling pathway to maintain the glucose homeostasis [19]. In addition, flavonoids may contribute to preserve the normal glycemia through the activation of the glucose uptake in insulin-sensitive tissues and the modulation of the hepatic glucose output and release [19]. In line with this, under physiological conditions in hepatic and renal cells, this mentioned effect was connected with both the activation of the IR-IRS-1 and-2-PI3K/AKT pathway and glucose uptake together with increased levels of GLUT-2, and the inhibition of glucose production [23,24]. Similarly, in adipocytes the incubation with different flavonoids stimulated the IR-IRS-PI3K/AKT route and the glucose uptake by inducing the translocation of GLUT-4 [25,26,27]. Cocoa procyanidins (PCs) have also been shown to mimic the insulin action in human primary skeletal muscle cells [28]. In particular, cocoa PCs stimulated the glycogen synthesis, the glucose uptake and the activity of the PI3K/AKT pathway. In the same way, the flavonoid epigallocatechin-3-O-gallate (EGCG) directly promoted the translocation of GLUT4 to the plasma membrane and improved the glucose uptake through the PI3K/AKT signaling pathway in L6 skeletal muscle cells [29].

All this points to a potential preventive activity for these phenolic compounds against different chronic diseases, including T2D and obesity, whilst in a healthy situation the insulin-like activity has been associated with a reinforcement of the hormone pathway. Accordingly, this review will focus on the molecular basis of the preventive activity of flavonoids related to insulin signaling in T2D and obesity.

## 4. Effects of Flavonoids on Insulin Signaling in T2D

T2D is the most widespread metabolic disease that affects more than 400 million adults worldwide and its prevalence has been increasing continuously, reaching epidemic proportions with a great social and health impact [30]. It is a complex metabolic disorder characterized by persistent elevated blood glucose due to the progressive insulin deficiency (beta-cell dysfunction) on the background of insulin resistance [31]. In diabetes, intrinsic genetic and epigenetic factors, as well as extrinsic factors, including circulating levels of lipids, glucose, or amino acids, can disrupt the insulin signaling network in insulin-sensitive tissues, leading to insulin resistance [32]. Numerous scientific evidences have revealed that flavonoids may contribute to prevent or ameliorate the insulin resistance in diabetes by their ability to modulate the insulin signaling pathway in classical target tissues such as liver, muscle, and adipose tissue. These effects have widely been reported from both *in vitro* and *in vivo* animal models (Table 2).

Liver plays a major role in maintaining the balance of glucose homeostasis; however, in diabetes, the ability of insulin to trigger downstream metabolic actions is impaired, leading to alterations in the hepatic metabolism [32]. In this regard, dietary flavonoids may enhance insulin sensitivity in diabetes by acting as insulin sensitizers. For instance, flavonoids such as rutin (23 μg/mL) and quercetin (6 μg/mL) have been demonstrated to overcome the high-glucose-induced insulin resistance in hepatic FL83B cells by promoting AKT phosphorylation, resulting in increased GLUT-2 translocation and glucose uptake [33]. Likewise, a cocoa flavonoid extract (1 µg/mL) and its main flavonoid epicatechin (EC) (10 µM) were able to diminish the insulin desensitization in glucose-induced insulin-resistant HepG2 cells by reducing IRS-1 serine phosphorylation and increasing tyrosine phosphorylated levels of IR, IRS-1 and IRS-2, and stimulating both the PI3K/AKT pathway and AMP-activated protein kinase (AMPK) [34]. Similar results were found with a swertisin rich flavonoid fraction both in insulin resistance hepatic cells [35] and in high fat diet (HFD)-fed and streptozotocin (STZ)-induced type 2 diabetic rats [36], as well as with the flavonoid tangeretin in (db/db) diabetic mice [37]. The hepatic insulin sensitizing effect of tangeretin [37] and cocoa flavonoids [55] has also been associated with their ability to suppress the MAPKs pathway. On the contrary, the flavonoid myricetin up-regulated p-IR, p-IRS1 and p-AKT in the liver of HFD-fed and STZ-induced type 2 diabetic rats by inhibiting the activity and expression of PTP1B, the tyrosine phosphatase that negatively regulates the insulin signal transduction [38]. Notably, the impaired insulin signaling in the liver promotes gluconeogenesis and suppresses glycogen synthesis [32]. In this sense, it has been shown that flavonoids can modulate several genes related to the glucose metabolism through the IRS/PI3K/AKT pathway [39,40,41]. For instance, the levels of glycogen and glycolytic enzymes were increased, whilst the expression of phosphoenolpyruvate carboxykinase (PEPCK) and glucose 6-phosphatase (G6Pase) was down-regulated in the liver of diabetic animals that were treated with isoquercetin (40 mg/kg bw) for 45 days [39]. Similarly, supplementation with a diet rich in cocoa flavonoids (10%) prevented the inactivation of the glycogen synthase kinase (GSK3)-β pathway and increased the phosphorylation levels of glycogen synthase (GS) in the liver of Zucker diabetic fatty (ZDF) rats, thus preserving the glycogen content [40]. In addition, cocoa supplementation decreased the expression levels of the gluconeogenic enzyme PEPCK and positively regulated those of glucokinase (GK), thus suppressing the hepatic glucose production. Likewise, treatment of db/db diabetic mice with a mulberry anthocyanin extract (50 and 125 mg/kg bw) reduced liver glycogen content, and that was consistent with changes in the phosphorylation of GSK3β and FOXO1, indicating that these alterations seemed to be related to gluconeogenesis suppression [41].

In diabetic conditions, the insulin signaling in the skeletal muscle is also compromised, leading to a lack of glucose utilization. The flavonoid myricitrin has been shown to activate the IRS-1/PI3K/AKT/GLUT4 signaling in both L6 muscle cells exposed to high glucose and in the soleus muscle of rats with T2D, improving the utilization of glucose in the diabetic milieu [42]. A study carried out with diabetic animals showed that the treatment with the flavonoid amentoflavone (20–40 mg/kg bw) for 8 weeks increased the level of phosphorylated AKT (Ser473) and the expression of GLUT-4 in the skeletal muscle, and that was associated with an improved peripheral glucose utilization and with a hypoglycemic effect [43]. Similarly, supplementation with a mulberry leaf flavonoid extract (2 g/kg bw) [44] or with the flavonoid phloretin (100 mg/kg bw) [45] upregulated IRS-1, PI3K and p-AKT levels in the skeletal muscle of type 2 diabetic rats. This activation was associated with the stimulation of GLUT-4 expression and its translocation, and with an increase in the insulin sensitivity. Interestingly, the combination of phloretin with metformin, the first-line antidiabetic drug, showed better results in the activation of the insulin signaling than each of those compounds alone [56]. Metformin is known to improve the insulin sensitivity through the modulation of AMPK, whereas flavonoids can exert their beneficial effects, at least partially, through the activation of the insulin pathway, indicating the interest of using flavonoids as adjuvants of classical therapies for the treatment of T2D. Other flavonoids such as chrysin [46] or an extract of mulberry anthocyanin [39] have also shown to recover the glycogen content in the skeletal muscle of diabetic animals through activation of the PI3K/AKT pathway.

Regarding the adipose tissue, the supplementation with an açai seed extract (rich in catechin, epicatechin and polymeric proanthocyanidins) improved the insulin sensitivity and reduced plasma glucose and lipid levels in type 2 diabetic animals together with an increase in p-AKT and GLUT-4 expression both in muscle and adipose tissue [47]. Similar results were found in ob/ob diabetic mice supplemented with the flavonoid nobiletin (200 mg/kg bw) [48]. Moreover, the oral administration of citrus fruit peel extracts and its constituting flavonoids naringin, naringenin, hesperidin and quercetin at a dose of 100 mg/kg bw for 4 weeks significantly recovered the suppressed mRNA expressions of GLUT-4 and IRβ-subunit in the adipose tissue of nicotinamide (NA)/streptozotocin-STZ/NA-induced type 2 diabetic rats [49,50]. Accordingly, the improved insulin sensitivity in the adipose tissue had potent anti-hyperglycemic and anti-hyperlipidemic effects in STZ/NA-induced diabetic rats.

Interestingly, flavonoids have also demonstrated their therapeutic potential for complications associated with diabetes by improving the impaired insulin signaling route in other non-classical targets such as the endothelium, kidney, and brain. In the endothelium, under hyperglycemic conditions, the deterioration of the IR/AKT/endothelial nitric oxide synthase (eNOS) pathway leads to a reduced nitric oxide (NO) production and the consequent endothelium-dependent relaxation of the aorta, which is closely associated with diabetic vascular complications [57]. It has been shown that catechin (50 mg/kg/day) supplementation in STZ-induced diabetic mice prevented diabetic endothelial dysfunction through the activation of endothelial PI3K and the subsequent activation of eNOS and NO generation [51]. In addition, insulin resistance in the kidney has also been related to the renal injury and the development of diabetic nephropathy. Indeed, impaired insulin signaling in the kidney has been associated with increased glucose uptake and apoptosis, which can significantly affect the kidney function [58]. In line with this, the flavonoid epicatechin (EC) and the microbial metabolite dihydroxyphenylacetic acid (DHPAA) prevented the dysfunction of renal cells treated with high glucose through the attenuation of the insulin signaling blockade and the modulation of glucose homeostasis via AKT and AMPK [52]. Likewise, supplementation with luteolin (10 mg/kg bw) for 4 weeks attenuated the renal damage in STZ-induced diabetic mice through increasing the phosphorylation of IR, PI3K and AKT in the kidney [53]. Moreover, insulin receptors are also expressed in the brain where insulin is essential for glucose homeostasis. Diabetic conditions alter the insulin-mediated PI3K-AKT signaling in neuronal cells and reduce the brain glucose metabolism, which in turn increases the risk of dementia, including Alzheimer disease, and can lead to the diabetic encephalopathy [59]. In this regard, flavonoids such as quercetin and naringenin, which are able to cross the blood–brain barrier, can regulate glucose transporters and other key components of the insulin signaling in the brain of STZ-induced diabetic rats, supporting a neuroprotective effect [54].

Collectively, the results obtained in *in vitro* and animal models indicate that dietary flavonoids can modulate the insulin signaling in peripheral tissues and, thus, alleviate the insulin resistance in T2D. A number of studies have also investigated the effects of the intake of flavonoid-rich foods in patients with T2D [60,61]; however, there are not clinical trials providing evidence on their effects on the insulin signaling at molecular level. Indeed, only a relatively small number of interventional studies have described the effect of flavonoids on the insulin sensitivity in patients with T2D, and the results obtained have been inconsistent (Table 3). In a randomized clinical trial conducted for one year on diabetic women, the daily intake of flavonoid-enriched chocolate containing 850 mg of flavanols and 100 mg of isoflavones resulted in a significant reduction of insulin resistance assessed by the homeostatic model assessment for insulin resistance (HOMA-IR) index [62]. Likewise, the consumption of a decaffeinated green tea extract providing a daily dose of 856 mg of EGCG for 16 weeks produced a significant reduction in HOMA-IR index in type 2 diabetic individuals [63]. In contrast, other studies have found that the acute and the short-term consumption of cocoa flavonoids had no effect on insulin sensitivity in diabetic patients [64,65,66,67]. Thus, supplementation with silybin-beta-cyclodextrin for 6 months tended to decrease the insulin resistance (HOMA-IR) in patients with T2D (differences were not significant) [68]. Similarly, in a double-blinded randomized crossover trial, diabetic patients supplemented with a grape seed extract (600 mg/day) for 4 weeks showed beneficial effects on lowering the fasting glucose, but effects on HOMA-IR index were not significant [69].

## 5. Effects of Dietary Flavanols on Insulin Signaling in Obesity

Obesity constitutes a worldwide health epidemy. According to WHO, 39% of adults (≥18 years-old) were overweight in 2016, and 13% were obese, and more importantly, 38 million children under the age of 5 were overweight or obese in 2019 [2].

Obesity is characterized by an abnormal fat accumulation in the white adipose tissue (WAT) and in peripheral important organs and tissues [70]. This disease is caused by an imbalance between the energy intake and expenditure, and the dysfunctionality of the adipose tissue is associated with an altered lipid metabolism, impaired adipose tissue expandability, and adipocyte hypertrophy [71]. All these abnormalities in the adipose tissue and peripheral organs are critical for the development of the insulin resistance [71], and have widely been studied at molecular level in insulin-responsive tissues and organs (i.e., adipose tissue, skeletal muscle and liver). In line with this, therapeutic strategies aimed at preventing insulin resistance should render protective effects against obesity. Accordingly, dietary interventions to introduce healthful food options, as increasing fruit and vegetable consumption, which are rich in flavonoids, could lead to body weight reduction and improved metabolic situation, and therefore, to the prevention of obesity [72]. At present, few clinical trials are available evaluating for health benefits related to the insulin signaling of flavonoids in obese patients (see below), and most of evidence comes from *in vitro* and *in vivo* works in which main target tissues for the disease, such as adipose tissue and other classical insulin-sensitive peripheral tissues (skeletal muscle and liver) have been studied (Table 4).

In cultured adipocytes, cyanidin-3-O-glucoside (C3G) (5–10 µM) prevented palmitic acid (PA)-induced insulin resistance [73]. Thus, C3G reduced the lipid accumulation and improved the insulin resistant condition by enhancing p-(Tyr895)-IRS-1, p85-PI3K and p-AKT values and decreasing p-(Ser307)-IRS-1 levels. In addition, C3G pre-treatment increased GLUT-1 values in 3T3-L1 cells [73]. Similarly, a hydroalcoholic extract rich in flavonoids from *Lampaya medicinalis* Phil. (HEL) was able to restore the IRS-1/AKT/AS160 pathway in cultured adipocytes [74]. Pre-incubation of cells with HEL (0.1 µg/mL) for 2 h followed by PA treatment (16 h) averted the decrease in p-(Tyr612)-IRS-1, p-AKT and p-AS160 levels, as well as the diminution in the glucose uptake.

In high-fat diet (HFD) fed rats, the administration of EGCG (3.2 g/kg food) for 16 weeks improved the insulin signaling pathway in the adipose tissue by decreasing p-IRS-1 values and increasing the levels of p85-PI3K and GLUT-4 [75]. Moreover, EGCG reduced the body weight, several metabolic parameters (i.e., FFA, fasting insulinemia, fasting glycemia, HOMA-IR, glucose infusion rate) and lowered the inflammatory condition present in obese HFD fed animals; indeed, the improvement of the insulin signaling in the adipose tissue was associated with the attenuated inflammation induced by EGCG [75]. Likewise, an alleviation in the insulin resistance situation was connected with a mitigation of the inflammation and an improvement of the redox status and metabolic condition, including lipid metabolism, in obese mice receiving nobiletin, an anthocyanidin-rich grape skin extract (GSE) or hydroxytyrosol [76,77,78]. Regarding the insulin signaling, nobiletin increased p-AKT and GLUT-4 levels in WAT of HFD fed mice [76], and GSE stimulated the IR-IRS-1-PI3K-AKT pathway, and enhanced GLUT-4 levels in the epididymal adipose tissue and gastrocnemius skeletal muscle of obese mice [77]. Accordingly, EGCG administration to triglyceride-infused rats ameliorated insulin resistance in WAT and soleus skeletal muscle by increasing p-AKT, p-AMPK and GLUT-4 levels, and decreasing p-(Ser307)-IRS-1, PKCθ translocation and oxidative stress [79]. Raspberry supplementation (rich in anthocyanins and ellagitannins) also improved the insulin sensitivity in HFD fed mice by modulating the insulin signaling pathway, as diminished p-(Ser307)-IRS-1 and p-(Ser676)-PKCθ levels, and increased p-AKT and GLUT-4 values in the inguinal WAT [80]. In addition, raspberry-rich diet decreased WAT hypertrophy, macrophage infiltration and inflammation, and induced beige adipogenesis through the enhancement of p-AMPK, peroxisome proliferator-activated receptor-gamma coactivator (PGC)-1α, sirtuin (SIRT)-1, uncoupling protein (UCP)-1, cytochrome c, and FNCD/irisin levels. 

Interestingly, opposite effects have been shown for genistein on insulin sensitivity under normal and inflammatory conditions in the adipose tissue [93]. Thus, in healthy mice after a glucose load genistein attenuated the IRS-1/AKT pathway, diminished GLUT-4 levels and enhanced p-AMPK values in the adipose tissue [93]. However, under a pro-inflammatory situation and after a glucose load reduced the levels of p-(Tyr)-IRS-1, p-AKT, GLUT-4 and IκB kinase (IKK), as well as enhanced values of p-(Ser307)-IRS-1 and p-AMPK were detected in the adipose tissue [93]. These results were explained by the key role of AMPK, which contributes to the anti-inflammatory effect of genistein, leading to the beneficial effect against insulin resistance.

Skeletal muscle is also an important target for evaluating the effects of flavonoids against insulin resistance during obesity [3,4]. Baicalin improved obesity-induced insulin resistance in C2C12 cultured myotubes and in mice fed with HFD [81]. Incubation of cells with this flavone (100, 200 and 400 µM) for 12 h increased p-AKT, p-Akt substrate of 160 kDa (AS160), GLUT-4, p-p38 and PGC-1α levels, as well as mRNA peroxisomal proliferator-activated receptor (PPAR)-γ and GLUT-1 values. Similarly, in HFD fed mice receiving 50 mg/kg baicalin daily, AKT/AS160/GLUT-4 and p38/PGC-1α/GLUT-4 pathways were stimulated, and contributed to reverse the insulin resistance, insulin intolerance and hyperglycemia in the skeletal muscle [81]. An improvement in the IRS-1/AKT/PI3K route, GLUT-4 levels and glucose utilization was observed when incubating L6 myotubes with phloretin previously exposed to PA (400 µM, 12 h), being this beneficial effect more pronounced when the natural phenol was combined with metformin [56]. In line with this, da Costa et al. [77] and Arunkumar et al. [82] have reported an upregulation in the IR-IRS-1-PI3K-AKT pathway, enhanced p-AMPK and GLUT-4 levels, and decreased values of p-ribosomal protein S6 kinase beta-1 (S6K1) in the skeletal muscle and epididymal adipose tissue of obese mice receiving a grape seed extract (GSE) and genistein, respectively. EGCG also reduced the insulin resistance through the improvement of the insulin signaling and redox status, the activation of GLUT-4 translocation and the AMPK pathway, as well as by the inhibition of PKCθ in the soleus muscle and WAT of rats infused for 48 h with a triglyceride emulsion that enhances FFA levels [79]. Likewise, green tea polyphenols attenuated the insulin resistance by stimulating the insulin signaling in the soleus muscle and improved the metabolic status in obese Zucker rats [83]. Mechanistically, the administration of green tea polyphenols prevented the inhibition of IRS-1/AKT signaling and increased GLUT-4 levels, which was associated with a diminished activity of PKCθ. All these molecular changes lead to an insulin-stimulated glucose uptake and decreased lipid accumulation. Flavanol-rich lychee extract (oligonol) supplementation also alleviated HFD-induced insulin resistance through the suppression of the inflammation in the tibialis anterior and gastrocnemius muscles and in the liver of mice [84]. Oligonol administration prevented the blockage of the insulin pathway in the gastrocnemius by upregulating the levels of IRS-1, p-(Tyr608)-IRS-1, p-AS160, and increasing p-AMPK and SIRT-1 values. These regulatory effects were related to an ameliorated insulin resistance and to a diminution of the intramuscular lipid content in the skeletal muscle. In the liver, oligonol also improved the insulin sensitivity through the decrease of p-GSK3 and p-phosphatase and tensin homolog (PTEN), and reduced the intracellular lipid content by inhibiting mammalian target of rapamycin (mTOR)/sterol regulatory element-binding protein 1 (SREBP-1) mediated lipogenesis [84]. In addition, oligonol decreased the adipocyte size (epididymal adipose tissue), as well as leptin and resistin levels through the downregulation of PPARγ [84]. All these beneficial modulatory effects contributed to improve the metabolic situation of the animals (glycemia, insulinemia, etc.) and to alleviate the inflammatory situation. On the contrary, in the skeletal muscle of HFD fed mice supplemented with quercetin, Stewart et al. [85] reported no effects on insulin resistance as p-AKT levels, PI3K values and activity, and triglyceride (TG) content remained unchanged in this tissue.

As mentioned above, the liver plays a key role in preserving glucose homeostasis, contributing to maintain the insulin sensitivity, also during obesity [3,4,7]. In line with this, aspalathin-enriched green rooibos (GRE) prevented PA-induced insulin resistance in C3A hepatic cells and improved the insulin sensitivity in obese rats [86]. GRE increased the phosphorylated levels of AKT and AMPK, as well as total levels of GLUT-2 *in vitro*. Moreover, GRE improved both glucose and lipid metabolism through the regulation of the FOXO1/carnitine palmitoyltransferase I (CPT1) route and by enhancing the uptake of both glucose and lipids in C3A cells [86]. A reversion in the blockage of the IRS-1/AKT/PI3K pathway, as well as enhanced GLUT-4 levels and glucose consumption were observed when incubating stimulated-PA BRL-3A cells with phloretin (50 µM) [56]. These beneficial results were more pronounced when this natural compound was combined with the anti-diabetic agent metformin [56]. Similarly, in obese insulin-resistant rats, GRE administration upregulated relevant genes involved in the insulin signaling pathway and glucose metabolism, namely *Insr, Irs1, Irs2, Pi3k* and *Ampk* [86]. Administration of a genistein-rich diet to high-fat-high-fructose fed mice improved the hepatic insulin resistance by abolishing the increased p-(Ser)-IRS-1 and -2 levels and p-S6K1, as well as through the stimulation of IR, IRS-1 and -2, PI3K, AKT and AMPK [87]. Moreover, in these mice, genistein reduced lipid accumulation through the downregulation of lipogenic genes and the upregulation of lipolytic genes. Likewise, EGCG and both aqueous and ethanolic-rich phenolic extracts from seeds of *Lepidium sativum* ameliorated the situation of insulin resistance in the liver of HFD fed rats by preventing the blockage of the insulin pathway (IR/IRS/AKT/mTOR/p70S6K) [88,89]; this improvement was connected with an amelioration of the pro-inflammatory and redox imbalance (enhancement in the enzymatic antioxidant activities). Accordingly, the prevention of both oxidative and endoplasmic reticulum stress in HFD-fed-mice induced by purple sweet potato color (anthocyanin) and hydroxytyrosol led to the attenuation of the hepatic insulin resistance [78,90]. Indeed, both natural substances restored the IRS-1/PI3K/AKT insulin signaling, as well as glucose and lipid metabolism in the liver of HFD-fed mice. An amelioration of the insulin resistance and endoplasmic reticulum stress was also observed in hepatic cultured cells exposed to palmitic acid and incubated with hydroxytyrosol [78]. In line with this, supplementation with *Vitis vinifera* L. grape skin extract (rich in anthocyanidins) for 12 weeks in HFD fed mice diminished insulin resistance, hyperglycemia and lipid accumulation in the liver [91]. Mechanistically, p-IRS-1, p-AKT, PI3K and GLUT-2 levels increased in animals receiving the skin grape extract, indicating an alleviation in the blockage of the insulin pathway induced by HFD. In addition, lipogenic proteins (SREBP1c) were upregulated and the lipolytic route (LKB1-AMPK) was downregulated, which was connected with the prevention of hepatic steatosis [91]. Blueberry supplementation also improved the insulin sensitivity and diminished insulin resistance in HFD fed rats [92]. These beneficial effects were related with decreased levels of p-(Ser307)-IRS-1 and HOMA-IR, and were found in conjunction with a reduced inflammation, changes in gut microbiota composition and improved gut integrity. On the contrary, quercetin-enriched diet initially (3 weeks of treatment) exacerbated the detrimental effects caused by the administration of a HFD in the liver of mice, whilst by the end of the study (8 weeks of treatment), both HFD fed animal groups were equally compromised [85].

In addition, in obesity, flavonoids exert beneficial effects related to the insulin signaling on non-classical insulin-targeted tissues, such as the endothelium and brain. Thus, luteolin (10 and 100 µM) improved the insulin sensitivity in Human umbilical vein endothelial (HUVEC) cells exposed to palmitic acid through the restoration of the p-IRS-1 values and the activation of the AKT/eNOS pathway, leading to an increased NO production [94]. In addition, luteolin reduced the PA-induced inflammation in HUVEC cells and increased the insulin-mediated endothelium-dependent relaxation in aortic rings from healthy rats. Likewise, purple sweet potato color supplementation (500 mg/kg/day for 11 weeks) alleviated the insulin resistance in the hippocampus of HFD fed mice, as mitigated the blockage of the IRS-1/PI3K/AKT route. This beneficial effect was also associated with a decreased glycemia, apoptosis and inflammatory condition, improved glucose tolerance and ameliorated cognitive impairment [95].

Most human trials aimed to evaluate the impact of flavonoids on obesity have analyzed clinical markers associated with this disease, such as body weight, body mass index (BMI) and lipid profile. A more reduced number of studies have reported the effects of these natural compounds on insulin resistance by providing indexes related to this situation during obesity, namely HOMA-IR and Quantitative Insulin Sensitivity Check Index (QUICKI) (Table 5). In this regard, in a randomized placebo-controlled cross-over study, participants received a controlled diet with blackberries (600 g/day containing 1500 mg flavonoids) or a calorie and carbohydrate matched amount of gelatin to be consumed within 12 h [96]. An improved HOMA-IR and insulin sensitivity together with an increased fat oxidation was reported. Similarly, Kosen-cha (a polymerized catechin-green tea, 1 L/day containing 1430 mg polyphenols), a pecan-rich diet (15% of total calories) and pomegranate juice (500 mL/day containing 842.5 mg polyphenols) reduced the insulin resistance (HOMA-IR) after the intervention [97,98,99]. This effect was accompanied by reduced values of insulinemia, glycemia and homeostasis model assessment of β-cell function (HOMA-B), and improved blood pressure (BP) and lipid profile. Nevertheless, other interventional studies have not reported a modification on the insulin resistance situation in overweight/obese subjects receiving flavanol-rich foods. Thus, in a randomized, controlled, crossover trial in which polyphenol-rich dark chocolate (20 g containing 500 mg polyphenols) was daily administered for 4 weeks, HOMA-IR and QUICKI indexes were unchanged [100]. A significant reduction in the body weight, systolic BP and diastolic BP was observed, whilst no differences were detected for the waist circumference and lipid profile [100]. Likewise, orange juice and green tea consumption did not improve HOMA-IR in overweight subjects [101,102]. Orange juice improved the lipid profile, the immune response, the inflammatory situation and the antioxidant capacity without modifying the body composition [101]. In contrast, the green tea reduced the body weight and insulinemia, and, as the orange juice, improved the lipid profile [102].

Interestingly, Ormazabal et al. [103] studied in visceral adipose tissue from normal and obese subjects the insulin responsiveness after protocatechuic acid incubation (100 µM, 24 h). This phenolic compound is easily generated by the metabolism of the microbiota after anthocyanin intake, and it was demonstrated that this compound was able to prevent the insulin resistance by increasing p-Tyr-IRS-1 and p-AKT levels in the visceral adipose tissue from obese volunteers. In addition, protocatechuic acid diminished the PTP1B activity and the inflammation (reduced p-p65 nuclear factor kappa B [NF-κB] and IL-6 levels) [103].

Altogether, in spite of the scarce number of studies evaluating the effect of flavonoids against insulin resistance during obesity at molecular level, the evidences suggest that these natural compounds may be promising agents for alleviating the alteration in the response of the hormone and other complications associated to this disease. Further studies are needed, especially in humans, to unravel the role of these natural compounds on insulin signaling during obesity.

## 6. Conclusions and Future Perspectives

**Figure 1:**
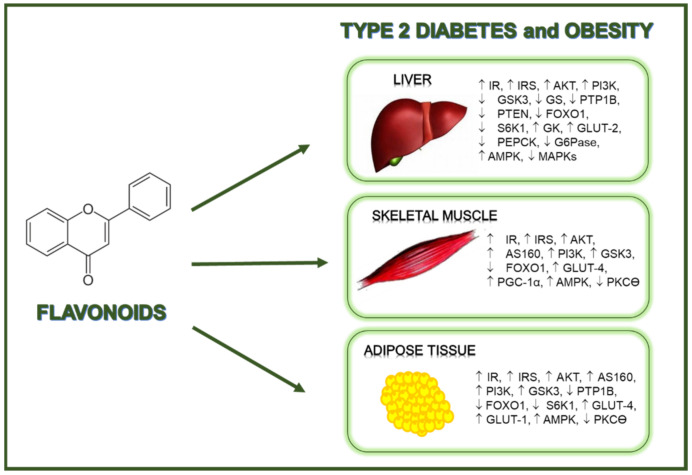
Flavonoids exert beneficial regulatory effects related to the prevention of diabetes and obesity.

Based on the studies presented in this review, the potential mechanisms of action induced by flavonoids in relation to the insulin signaling have been evidenced in *in vitro* and *in vivo* models of T2D and obesity. Overall, flavonoids exert beneficial effects against these pathologies through the modulation of key elements of the insulin signal transduction pathway and the regulation of glucose transport, being these actions associated with a reduced insulin resistance, and both improved insulin sensitivity and glucose tolerance (Figure 1). However, it is interesting to note that at present, very few human interventional clinical trials have reported some favorable insulin-sensitizing effects, and even a lower number of works have studied the impact of these flavonoids on the insulin route at molecular level. Therefore, further investigations, and especially more well-controlled interventional human studies, are necessary to truly estimate the potential of flavonoids in terms of insulin-mimetic and insulin-sensitizing effects, elucidation of the molecular mechanism and targets of these natural compounds in relation to the insulin signaling pathway, and definition of optimal doses. All this knowledge would allow disclosing how to obtain a positive effect from these natural compounds to prevent, delay or contribute to the treatment of T2D and obesity.

## Figures and Tables

**Table 1:** Basic chemical structure of flavonoids and flavonoids subgroups with representative compounds and main dietary sources.

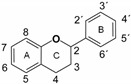		
Flavonoid Subgroups	Representative Compounds	Main Dietary Sources
FLAVONES 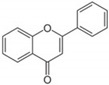	Luteolin Apigenin Acacetin Chrysin Diosmetin	Chamomile, parsley, tea, fenugreek seed, oregano, peppermint
FLAVANONES 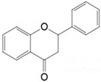	Hesperidin Naringenin Isosakuratenin Eriodictyol	Orange, grapefruit, lime, mandarin, bergamot, lemon, chinotto, tangor and tangerine
FLAVONOLS 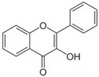	Quercetin Myricetin Kaempherol Rutin	Onion, blueberry, apple, broccoli, tomato, tea, red wine
FLAVANOLS 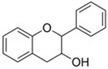	Epicatechin Catechin Epigalocatechin Epigalocatechin-3-gallate	Cocoa, red wine, green tea, red grape, berries, apple, cherry, apricot, peach
ISOFLAVONES 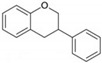	Genistein Daidzein	Soya, legumes
ANTHOCYANIDINS 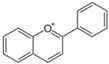	Cyanidin Malvidin Delphinidin Pelargonidin Peonidin	Berries, cherry, grape, pomegranate, red onion, red wine

**Table 2:** Effects of flavonoids on insulin signaling in classical and no-classical target tissues in T2D ^a^.

Cell/Animal Model	Treatment	Effect on Insulin Signaling	Main Metabolic Outcomes	Reference
**Liver**				
Mouse FL83B cells	Rutin (23 μg/mL) or quercetin (6 μg/mL) with glucose (30 mM) during 48 h	↑p-AKT, ↑AMPK, ↓PTP1B, ↑GLUT-2	↑Glucose uptake	[33]
Human HepG2 cells	Epicatechin (10 µM) or cocoa flavonoid extract (1 µg/mL) during 24 h and glucose (30 mM) for additional 24 h	↓p-(Ser)-IRS-1, ↑IR, ↑IRS-1, ↑IRS-2, ↑p-AKT, ↑p-GSK3, ↑p-AMPK, ↑GLUT-2, ↓PEPCK	↑Glucose uptake ↑Glycogen levels	[34]
Human HepG2 cells	High glucose (25 mM) + insulin (2.5 µg/mL) for 24 h and swertisin rich flavonoid extract (50 µg/mL) for additional 24 h	↑IRS-1, ↑AKT-2, ↑GLUT-4	↑Glucose uptake	[35]
HFD-fed and STZ-induced type 2 diabetic rats	Swertisin rich flavonoid extract (25, 50 and 100 mg/kg body weight), 28 days	↑IRS-1, ↑GLUT-2, ↑GLUT-4, ↑GK	↓Blood glucose ↑Glucose tolerance ↓HOMA-IR, ↓TG ↑Glycogen levels	[36]
(db/db) diabetic mice	Tangeretin (50 mg/kg bw), 30 days	↑IR, ↑AKT, ↑GSK3	↓Blood glucose ↑Glucose tolerance ↓HOMA-IR	[37]
HFD-fed and STZ-induced type 2 diabetic rats	Myricetin (20 mg/kg bw), 4 weeks	↑p-IR, ↑p-IRS1, ↑p-AKT, ↓PTP1B enzyme activity	↓Blood glucose, ↓HbA1c ↓HOMA-IR ↓TG, ↓LDL, ↑HDL	[38]
STZ-induced diabetic rats	Isoquercetin (40 mg/kg bw), 45 days	↑IR, ↑IRS-1, ↑IRS-2, ↑AKT ↑GLUT-2, ↑GK, ↓G6Pase, ↓PEPCK	↓Blood glucose ↑Glucose tolerance ↑Glycogen levels	[39]
Zucker diabetic fatty rats [ZDF/crl-lepr (fa/fa)]	10% cocoa rich diet, 9 weeks	↓p-(Ser)-IRS-1, ↑p-GSK3, ↓p-GS, ↑GLUT-2 ↓PEPCK, ↑GK	↓Blood glucose, ↓HbA1c ↑Glucose tolerance ↓HOMA-IR ↓ Glycogen levels	[40]
(db/db) diabetic mice	Mulberry anthocyanin extract (50 and 125 mg/kg bw), 8 weeks	↑AKT, =p-GSK3, ↑FOXO1	↓Blood glucose ↑Glucose tolerance, ↓HOMA-IR, ↓TG, ↓LDL ↓Glycogen levels	[41]
**Muscle**				
HFD-fed and STZ-induced type 2 diabetic rats	Myricetin (300 mg/kg bw), 28 days	↑p-IRS-1, ↑p85-PI3K, ↑GLUT-4	↓Blood glucose ↓HOMA-IR, ↑HOMA-β ↓TG, ↓LDL, ↑HDL	[42]
HFD-fed and STZ-induced type 2 diabetic mice	Amentoflavone (20–40 mg/kg, bw), 8 weeks	↑AKT, ↑GLUT-4	↓Blood glucose ↑Glucose tolerance ↓TG, ↓LDL, ↑HDL	[43]
HFD-fed and STZ-induced type 2 diabetic rats	Flavonoids mulberry leaf extract (2 g/kg bw), 4 weeks	↑IRS-1, ↑p85-PI3K, ↑AKT, ↑GLUT-4	↓Blood glucose ↑Glucose tolerance ↓HOMA-IR, ↓TG, ↓LDL	[44]
High fat and high sugar fed and STZ-induced type 2 diabetic rats	Phloretin (100 mg/kg, bw), 4 weeks	↑IRS-1, ↑p85-PI3K, ↑AKT, ↑GLUT-4	↓Blood glucose ↑Glucose tolerance ↓HOMA-IR ↓TG, ↓LDL, ↓HDL, ↓FFA	[45]
High fat and sucrose fed-induced type-2 diabetic rats	Chrysin (25–200 mg/Kg. bw), 30 days	↑IR, ↑IRS-1, ↑AKT, ↑GLUT-4	↓Blood glucose ↑Glucose tolerance ↓TG, ↓LDL, ↑HDL, ↓FFA ↑Muscle glycogen levels	[46]
(db/db) diabetic mice	Mulberry anthocyanin extract (50 and 125 mg/kg bw)	↑AKT, ↑p-GSK3β	↓Blood glucose ↑Glucose tolerance ↓HOMA-IR ↑Muscle glycogen levels	[41]
**Adipose**				
HFD-fed and STZ-induced type 2 diabetic rats	Açai seed extract (200 mg/kg bw)	↑p-AKT, ↑GLUT-4	↓Blood glucose, ↓HbA1c ↓HOMA-IR, ↑ HOMA-β ↓TG, ↓LDL, ↑HDL	[47]
(ob/ob) obese diabetic mice	Nobiletin (200 mg/kg bw), 5 weeks	↑p-AKT, ↑GLUT-4, ↑GLUT-1	↓Blood glucose ↑Glucose tolerance ↓HOMA-IR	[48]
NA/STZ-induced type 2 diabetic rats	Navel orange peel hydroethanolic extract, naringin, and naringenin (100 mg/kg bw), 4 weeks	↑IR, ↑GLUT-4,	↓Blood glucose ↑Glucose tolerance ↓TG, ↓LDL, ↑HDL, ↓FFA	[49]
NA/STZ-induced type 2 diabetic rats	*C. reticulata* fruit peel hydroethanolic extract, hesperidin, and quercetin (100 mg/kg bw), 4 weeks	↑IR, ↑GLUT-4,	↓Blood glucose ↑Glucose tolerance ↓HOMA-IR, ↑ HOMA-β ↓TG, ↓LDL, ↑HDL, ↓FFA	[50]
**Others tissues**				
STZ-induced diabetic rats	Catechin (50 mg/kg bw), 3 weeks	↑PI3K in endothelium	↓Blood glucose ↑Aortic nitrite/nitrate concentration. ↑Endothelium-dependent relaxation	[51]
Rat NRK-52E renal cells	Epicatechin (10 µM) or DHPAA (10 µM) during 2 h and glucose (30 mM) for additional 22 h	↑IR, ↑p-IR, ↑p-GSK3, ↓GS, ↓PEPCK in renal cells	↑Glucose uptake ↓Glucose production	[52]
STZ-induced diabetic mice	Luteolin (10 mg/kg bw), 4 weeks	↑IR, ↑PI3K, ↑AKT in kidney	↓Blood glucose, ↓TG, ↓LDL ↑Glucose tolerance ↓serum and urine levels of creatinine and uric acid	[53]
STZ-induced diabetic rats	Quercetin (0.1%) or Naringenin (0.05%), 2 months	↑IRS-1, ↑PI3K, ↑AKT ↑GLUT-1, ↑GLUT-3 in brain	↓Blood glucose	[54]

^a^ The arrow indicates an increase (↑) or decrease (↓) in the levels of the different parameters analyzed. **AKT**: protein kinase B; **AMPK**: AMP-activated protein kinase; **DHPAA**: dihydroxyphenylacetic acid; **FFA**: free fatty acids; **FOXO1**: Forkhead Box protein O1; **GLUT**: glucose transporter; **GK**: glucokinase; **GS**: glycogen synthase; **GSK3**: glycogen synthase kinase-3; **G6Pase**: glucose 6 phosphatase; **HbA1c**: glycated hemoglobin; **HDL**: high density lipoprotein; **HFD**: high fat diet; **HOMA-β**: homeostatic model assessment of beta cell; **HOMA-IR**: homeostatic model assessment of insulin resistance; **IRS**: insulin receptor; **IRS**: insulin receptor substrate; **LDL**: low density lipoprotein; **NA**: nicotinamide; **PEPCK**: phosphoenolpyruvate carboxykinase; **PI3K**: phosphoinositide 3-kinase; **PTP1B**: protein tyrosine phosphatase 1B; **STZ**: streptozotocin; **TG**: triglycerides.

**Table 3:** Human interventional trials of the effects of flavonoid and flavonoid-rich food intake on insulin resistance in diabetes ^a^.

Type of Study	Number of Participants	Treatment	Effect on Metabolism	Reference
RCDB, parallel	93♀ (post-menopausal (type 2 diabetic patients)	850 mg flavanols and 100 mg isoflavones/day, 1 year	=Glycemia, =HbA1c, ↓Insulinemia, ↓HOMA-IR, ↓LDL, =HDL	[62]
RCDB, parallel	68 (35♂ + 33♀) (type 2 diabetic patients)	1500 mg of green tea extract (856 mg of ECGC)/day, 16 weeks	=Glycemia, ↓Insulinemia, ↓HOMA-IR, ↑HDL, =LDL, =TG	[63]
RCDB, crossover	12 (3♂ + 9♀) (hypertensive type 2 diabetic patients)	83.6 mg of cocoa flavanols, acute	=Glycemia, =Insulinemia, =HOMA-IR, =LDL, =HDL, =TG	[67]
RCDB, crossover	18 (4♂ + 14♀) (type 2 diabetic patients)	Cocoa beverage (960 mg polyphenols), acute	=Glycemia, ↑Insulinemia, =HOMA-IR, ↑HDL, =LDL, =TG	[64]
RCDB, parallel	35 (18♂ + 17♀) (hypertensive type 2 diabetic patients)	83.6 mg of cocoa flavanols/day, 12 weeks	=Glycemia, =HbA1c, =Insulinemia, =HOMA-IR, =LDL, =HDL, =TG	[65]
RCDB, crossover	12 (7♂ + 5♀) (hypertensive type 2 diabetic patients)	Dark chocolate (450 mg flavanols)/day, 8 weeks	=Glycemia, =HbA1c, =Insulinemia, =HOMA-IR, ↑HDL, =LDL	[66]
RCDB, parallel	42 (20♂ + 22♀) (type 2 diabetic patients)	135 mg silybin/day, 6 months	↓Glycemia, =Insulinemia, =HOMA-IR, =HDL, ↓TG	[68]
RCDB, crossover	32 (16♂ + 16♀) (type 2 diabetic patients)	Grape seed extract (600 mg of flavonoids)/day, 4 weeks	=HOMA-IR, =HDL, =TG, ↓TC	[69]

^a^ The arrow indicates an increase (↑) or decrease (↓) in the levels of the different parameters analyzed, “=” symbol designates unchanged parameters. **EGCG**: epigallocatechin-3-gallate; **HbA1c**: hemoglobin glycosylated; **HDL**: high-density lipoprotein; **HOMA-IR**: Homeostasis Model Assessment of Insulin Resistance; **LDL**: low-density lipoprotein; **RCDB**: randomized controlled double-blinded; **TC**: total cholesterol; **TG**: triglycerides.

**Table 4:** Effects of flavonoids on insulin signaling in classical target tissues in obesity ^a^.

Cell/Animal Model	Treatment	Effect on Insulin Signaling	Main Metabolic Outcomes	Reference
**Adipose**				
Mouse 3T3L1 cells	Cyanidin-3-O-glucoside (5–10 μM, 24 h) followed by palmitic acid (500 μM, 24 h)	↓p-(Ser)-IRS-1, ↑p-(Tyr)-IRS-1, ↑p85-PI3K, ↑pAKT, ↑GLUT-1	↓Insulin resistance ↓Lipid accumulation	[73]
Mouse 3T3L1 cells	Hydroethanolic extracts of lampaya (0.1 µg/mL) for 2 h followed by palmitic acid (650 µM) during additional 16 h	↑p-(Tyr)-IRS-1, ↑p-AKT, ↑pAS160	↑Glucose uptake	[74]
HFD-fed rats (40% fat)	EGCG (3.2 g/kg food), 16 weeks	↓p-(Ser)-IRS-1, ↑p85-PI3K, ↑GLUT-4	↓Glycemia, ↑GIR ↓Insulinemia, ↓HOMA-IR ↓BW, ↓FFA	[75]
HFD-fed mice (61% fat)	Nobiletin (10 and 100 mg/kg bw), 5 weeks	↑p-AKT, ↑GLUT-4	↑Glucose tolerance ↓BW, ↓TG	[76]
HFD-fed mice (60% fat)	Anthocyanidin-rich *Vitis vinifera* L. grape skin extract (200 mg/kg bw), 12 weeks	↑IR, ↑p-(Tyr)-IRS, ↑PI3K, ↑pAKT, ↑GLUT-4	↓Glycemia ↓Insulinemia ↓HOMA-IR ↓BW, ↓lipidemia	[77]
HFD-fed mice (45% fat)	Hydroxytyrosol (20 mg/kg bw), 10 weeks	↓p-(Ser)-IRS-1, ↑p-AKT, ↑GLUT-4, ↓p-JNK	↓Glycemia ↓Insulinemia ↓HOMA-IR ↓Insulin resistance ↑Glucose tolerance	[78]
Lipid-injected rats (infusion 20% intralipid plus 20 U/mL heparin at 5.5 mL/min) (co-injection)	EGCG (5 and 10 mg/kg bw), 48 h	↓p-(Ser)-IRS-1, ↑p-AKT, ↑p-AMPK, ↑GLUT-4 translocation, ↓p-PKCθ translocation	↓Glycemia, ↑GIR ↓Insulinemia, ↓FFA	[79]
HFD-fed mice (35% fat)	5% Freeze dried raspberry-rich diet, 12 days	↓p-(Ser)-IRS-1, ↑p-AKT, ↓p-(Ser)-PKCθ, ↑GLUT-4, ↓p-p38, ↑p-ERK, ↑p-AMPK	↓Insulin resistance	[80]
**Muscle**				
Mouse C2C12 cells	Baicalin (100, 200 and 400 µM, 12 h)	↑p-AKT, ↑p-AS160, ↑p-p38, ↑GLUT-4, ↑mRNA GLUT-1	↑Glucose uptake	[81]
HFD-fed mice (59% fat)	Baicalin (50 mg/kg bw), 16 weeks	↑p-AKT, ↑p-AS160, ↑p-p38, ↑GLUT-4, ↑mRNA GLUT-1	↓Glycemia ↓Insulinemia ↓HOMA-IR ↑Glucose tolerance, ↓BW	
Rat L6 cells	Phloretin (50 μM, 24 h) followed by palmitic acid (400 μM, 12 h)	↑IRS-1, ↑p-AKT, ↑PI3K, ↑GLUT-4	↑Glucose utilization	[56]
HFD-fed mice (60% fat)	Anthocyanidin-rich *Vitis vinifera* L. grape skin extract (200 mg/kg b.w.), 12 weeks	↑IR, ↑p-(Tyr)-IRS, ↑PI3K, ↑p-AKT, ↑GLUT-4, ↑p-AMPK	↓Glycemia ↓Insulinemia ↓HOMA-IR ↓BW, ↓lipidemia	[77]
High fat (20%)-high fructose (45%) diet	Genistein (1 mg/kg bw/day), 60 days	↑p-(Y)-IR, ↑p-(Y)-IRS-1, ↓p-(Ser)-IRS-1, ↑p85-PI3K, ↑p-AKT, ↑GLUT-4, ↑p-AMPK	↓Glycemia ↓Insulinemia ↓HOMA-IR ↓QUICKI ↓BW	[82]
Lipid-injected rats (infusion 20% intralipid plus 20 U/mL heparin at 5.5 mL/min) (co-injection)	EGCG (5 and 10 mg/kg bw), 48 h	↓p-(Ser)-IRS-1, ↑p-AKT, ↑p-AMPK, ↑GLUT-4 translocation, ↓p-PKCθ translocation	↓Glycemia, ↑GIR ↓Insulinemia ↓FFA	[79]
HFD-fed Zucker fatty rats (59% fat)	Green tea polyphenols (70.9% EGCG + 1.7% EGC + 7.4% ECG, 19.3% EC) (200 mg/kg bw), 8 weeks	↑total GLUT-4, ↑GLUT-4 translocation, ↓p-(Ser)-IRS-1, ↑p-AKT, =PKCβ2, =PKCε, ↓p-PKCθ translocation, ↓PKCξ	↓Glycemia ↓Insulinemia ↓HOMA-IR ↑Glucose tolerance ↓Insulin resistance ↓BW, ↓visceral adiposity ↓Lipid accumulation	[83]
HFD-fed mice (60% fat)	Oligonol (20 and 200 mg/kg bw), 12 weeks	=IRβ, ↑IRS-1, ↑p-(Tyr)-IRS-1, ↑p-AS160, ↑p-AMPK	↓Glycemia ↓Insulinemia ↑Glucose tolerance ↓BW, ↓TG ↓lipid accumulation	[84]
HFD-fed mice (45% fat)	1.2% quercetin-rich diet, 8 weeks	=pAKT, =PI3K	=Glycemia ↓Insulinemia =GIR =peripheral glucose uptake =BW =TG, =NEFA =short-fatty acylcarnitines ↑long-fatty acylcarnitines	[85]
**Liver**				
Human C3A cells	Aspalathin-enriched green rooibos (10 µg/mL, 30 min) + palmitic acid (0.75 mM, 3 h)	↑p-AKT, ↑GLUT-2, ↑p-AMPK, ↓FOXO1	↑Glucose uptake ↑Fatty acid uptake ↑Glycerol release ↓Lipid accumulation	[86]
High fat (40%)-high-sugar (44%) diet fed rats (obese insulin-resistant rats)	Aspalathin-enriched green rooibos (32, 97 and 195 mg/kg), 12 weeks	↑*Ins*, ↑*Irs1*, ↑*Irs-2*, ↑*Pi3k*, ↑*Ampk*, *=glut-2*	=Glycemia ↓Insulinemia ↓HOMA-IR	
Rat BRL3A cells	Phloretin (50 μM, 24 h) followed by palmitic acid (200 μM, 12 h)	↑IRS-1, ↑p-AKT, ↑PI3K, ↑GLUT-4	↑Glucose consumption	[56]
High fat (20%)-high fructose (45%) diet	Genistein (1 mg/kg bw/day), 45 days	↑IRS-1, ↑IRS-2, ↑p-AKT, ↑p-AMPK, ↓p-S6K1	↓Glycemia ↓Lipid accumulation ↓FFA, ↓TG, ↓Cho ↓BW	[87]
HFD fed rats (60% fat)	EGCG (3.2 g/kg bw), 16 weeks	↑p-(Y)-IRS-1, ↑IRS-2, ↑PI3K, ↑AKT	=Glycemia ↓Insulinemia ↓HOMA-IR, ↑ GIR ↓FFA, ↓TG	[88]
HFD fed rats (59% fat)	*Lepidium sativum* ethanol extracts (200 and 400 mg/kg bw) and *Lepidium sativum* aqueous extract (200 mg/kg bw), 8 weeks	↑p-IR, ↑p-AKT, ↑p-mTOR, ↓p-70S6K1	↓Glycemia ↓Insulinemia ↓HOMA-IR, ↑ HOMA-B ↓total lipids, ↓Cho, ↓TG, ↓HDL, ↓LDL	[89]
HFD fed mice (60% fat)	Purple sweet potato color (700 mg/kg bw), 20 weeks	↓p-(Ser)-IRS-1, ↑p110β-PI3K, ↑p85α-PI3K, ↑p-AKT, ↑p-GSK3β	↓Glycemia ↑Glucose tolerance ↓Insulin resistance ↓Lipid accumulation ↓FFA, ↓Cho, ↓TG ↓BW	[90]
HFD-fed mice (45% fat)	Hydroxytyrosol (20 mg/kg bw), 10 weeks	↓p-(Ser)-IRS-1, ↑p-AKT, =GLUT-2	↓Glycemia ↓Insulinemia ↓HOMA-IR ↓Insulin resistance ↑Glucose tolerance	[78]
Human HepG2 cells	Hydroxytyrosol (50 and 100 µM)+palmitic acid (100 µM), 1 h	↑p-(Tyr)-IRS-1, ↑p-AKT	↓Insulin resistance	
HFD fed mice (60% fat)	*Vitis vinifera* skin extract (200 mg/kg bw), 12 weeks	↑p-IRS-1, ↑p-AKT, ↑PI3K, ↑GLUT-2, ↑p-AMPK	↓Glycemia ↓Insulinemia ↓HOMA-IR ↓Glycogen content ↓Lipid accumulation ↓Cho, ↓TG, ↓HDL, ↓LDL	[91]
HFD-fed rats (45% fat)	10% Freeze dried blueberry-rich diet, 8 weeks	↓p-(Ser)-IRS-1	=Glycemia =Insulinemia ↓HOMA-IR =Glucose tolerance =BW	[92]
HFD-fed mice (60% fat)	Oligonol (20 and 200 mg/kg bw), 12 weeks	=p-AKT, ↓p-GSK3α, ↓p-(Ser)-PTEN, ↓p-mTOR, ↑p-AMPK	↓Glycemia ↓Insulinemia ↑Glucose tolerance ↓BW, ↓TG ↓Lipid accumulation	[84]
HFD-fed mice (45% fat)	1.2% quercetin-rich diet, 8 weeks	=p-AKT, =PI3K	=Glycemia ↓Insulinemia =GIR =peripheral glucose uptake =BW =TG, =NEFA ↑short-fatty acylcarnitines ↓long-fatty acylcarnitines	[85]

^a^ The arrow indicates an increase (↑) or decrease (↓) in the levels of the different parameters analyzed, “=” symbol designates unchanged parameters. **AKT**: protein kinase B; **AMPK**: AMP-activated protein kinase; **AS160**: Akt substrate of 160 kDa; **BW**: body weight; **Cho**: cholesterol; **EC**: epicatechin; **ECG**: epicatechin gallate; **EGC**: epigallocatechin; **EGCG:** epigallocatechin-3-gallate; **ERK**: extracellular signal-regulated kinases; **FFA**: free fatty acid; **FOXO1**: Forkhead Box protein O1; **GIR**: glucose infusion rate; **GLUT**: glucose transporter; **GK**: glucokinase; **GSK3**: glycogen synthase kinase; **HDL**: high-density lipoprotein; **HFD**: high fat diet; **HOMA-B**: homeostasis model assessment of β-cell function; **HOMA-IR**: homeostasis model assessment of insulin resistance; **IR**: insulin receptor; **IRS**: insulin receptor substrate; **JNK**: Jun N-terminal kinase; **LDL**: low-density lipoprotein; **mTOR**: mammalian target of rapamycin; **NEFA**: non-esterified fatty acid; **OGTT**: oral glucose tolerance test; **p38**: p38 mitogen-activated protein kinase (MAPK); **PI3K**: Phosphoinositide 3-kinase; **PKC**: protein kinase C; **PTEN**: phosphatase and tensin homolog; **QUICKI**: quantitative sensitivity check index; **p70-S6K1**: ribosomal protein S6 kinase beta-1; **TG**: triglycerides.

**Table 5:** Human interventional trials of the effects of flavonoid and flavonoid-rich food intake on insulin resistance during obesity ^a^.

Type of Study	Number of Participants	Treatment	Effect on Metabolism	Reference
RC, crossover	27 ♂ (BMI > 25 kg/m^2^, 53–63 years)	600 g blackberries (1500 mg flavonoids), acute (12 h)	=Glycemia, =Insulinemia, HOMA-IR, ↓HOMA-B, ↓AUC in GTT for insulin ↓respiratory quotient, ↓AUC for NEFA, ↓TG	[96]
Prospective	6 (4♂ + 2♀), (BMI = 25–35 kg/m^2^, ≥45 years)	Kosen-cha (1 L/day, 14,300 mg polyphenols), 12 weeks	=Glycemia, ↓HOMA-IR ↓BW, ↓BMI, ↓WC, ↓TG	[97]
RCB, crossover	26 (21♂ + 5♀), (BMI > 25 kg/m^2^, 38–58 years)	Pecan-rich diet (15% of total calories), 12 weeks	↓Glycemia, ↓Insulinemia, ↓HOMA-IR, ↓HOMA-B ↓VLDL, ↓LDL, ↓HDL, ↓TG	[98]
RC, crossover	28 (12♂ + 16♀), (BMI = 25–35 kg/m^2^, 40–65 years)	Pomegranate juice (500 mL, 1685 mg polyphenols/L), 4 weeks	↓Insulinemia, ↓HOMA-IR =BW, =BMI	[99]
RCSB, crossover	42 adult women (25 overweight [BMI ≥ 25 kg/m^2^] + 21 controls, [BMI = 18–24.9 kg/m^2^])	Dark chocolate (20 g, 500 mg polyphenols), 4 weeks	=Insulinemia, =HOMA-IR, ↓QUICKI ↓BW, =WC	[100]
RCDB, parallel	46 adult women (13 overweight + 8 obese, [BMI ≥ 25 kg/m^2^] + 21 controls, [BMI = 25 kg/m^2^])	Orange juice (750 mL/day, 135 mg flavonoids/L), 8 weeks	=Glycemia, =Insulinemia, =HOMA-IR. ↓Total Cho, ↓LDL, =HDL. =BMI, =body fat mass, =body mass, =WC	[101]
RCDB, parallel	54 breast cancer survivors (BMI = 25–40 kg/m^2^, 18–80 years)	Green decaffeinated tea (960 mL/day, 235.64 mg catechin and 128.84 mg EGCG), 6 months	=Glycemia, =Insulinemia, =HOMA-IR. ↓LDL, ↑HDL, =TG ↓BW, ↓BMI, ↓fat mass, ↓lean mass, ↓WC, ↓hip circumference	[102]

^a^ The arrow indicates an increase (↑) or decrease (↓) in the levels of the different parameters analyzed, “=” symbol designates unchanged parameters. **AUC**: area under the curve; **BMI**: body mass index; **BW**: body weight; **Cho**: cholesterol; **EGCG**: epigallocatechin-3-gallate; **HDL**: high-density lipoprotein; **HOMA-B**: homeostasis model assessment of β-cell function; **HOMA-IR**: homeostasis model assessment of insulin resistance; **LDL:** low-density lipoprotein; **NEFA**: non-esterified fatty acid; **QUICKI**: quantitative sensitivity check index; **RC**: randomized controlled; **RCB**: randomized controlled blinded; **RCDB**: randomized controlled double-blinded; **RCSB**: randomized controlled single-blinded; **TG**: triglycerides; **VLDL**: very low-density lipoprotein; **WC**: waist circumference.
